# Supplementary material for: High correlation between detection of dengue IgG from dried blood spots and serum using an indirect IgG ELISA assay: A validation study in Fortaleza, Brazil
Source: PLoS Negl Trop Dis. 2025 Feb 28;19(2):e0012880. doi: 10.1371/journal.pntd.0012880 (PMC11893133; doi:10.1371/journal.pntd.0012880)
Supplement: Attachment — Submitted filename: Response to reviewers 2.docx [file pntd.0012880.s003.docx]

**Response to reviewer comments and questions**

**1. Reviewer #2:** I would prefer percentages in table 2 to be 'row percentages' rather than 'column percentages'. I believe this would make it more immediately interpretable. Also add a 'total' column on the right. Not an essential change.

**Authors’ response:** We have made these changes (pages 11 and 12).

**2.** **Reviewer #2:** It is still not clear to me what each of the horizontal dotted lines are, in Figure 4. I think further explanation is needed, or they could be removed.

**Authors’ response:** We apologize for the continued confusion regarding Figure 4. The horizontal dotted lines are the 95% confidence intervals (95% CIs) for the Bland-Altman plot; there are three sets of CIs since there is one set each for the Bland-Altman mean of differences, the lower limit of agreement (LOA), and the upper LOA. We have added additional clarification in the text for this Figure, which can be found on page 13, lines 225 to 229: “The central dashed line is the Bland-Altman mean of differences, with adjacent horizontal dotted lines indicating the corresponding 95% CI. The lower dashed line is the lower LOA, while the upper dashed line is the upper LOA. The 95% CI of each LOA is represented by adjacent horizontal dotted lines.” Along with the values for the Bland-Altman plot given in the text, including the CIs, we hope that this will be clear for readers.

**3.** **Reviewer #2:** It is clear from Figure 3, Figure 4, and from your analysis of discrepant results that DBS is likely giving higher IVs than serum at the low end of the assay range (i.e. below 1.5, which includes the serological cutoff at 0.9-1.1). This is mentioned in the discussion BUT I don't think the potential impact of this observation is adequately discussed yet. In this study of adults, IGG was mostly detected strongly (high IV) or not detected (low IV). There were very few results near to the cutoff. Therefore, the authors observed very good agreement between DBS and serum. However, if there were more samples closer to the cutoff, this likely would not have been the case. Children, who for many reasons are generally targeted in dengue serosurveys (and who are specifically mentioned in the intro to this paper) may have a higher proportion of 'monotypic' antibody responses (i.e. after only one previous infection) and may have lower concentrations of IgG. In this group, DBS could over-call seropositivity more (compared to adults).

Were there any experiments using DBS elude at 1:8? It looks as if further dilution of the elute may have resulted in IV equivalence when it was 0.9-1.1 , which I would argue is the 'optimal' target.

Anyway, I do think this needs to be set out a bit more clearly, as any subsequent researchers/studies which use this DBS protocol would need to be careful with any DBS results which are around the cutoff (and crucially what proportion of the results are near the cutoff).

**Authors’ response:** Thank you for raising this thoughtful comment. While we did not test the 1:8 dilution ourselves, we agree that these are important points to be clarified for readers, and have added additional discussion of this topic on page 15, lines 248 to 251. We hope that this will help improve the clarity of the discussion for others who wish to follow a similar protocol.
