# Supplementary material for: High correlation between detection of dengue IgG from dried blood spots and serum using an indirect IgG ELISA assay: A validation study in Fortaleza, Brazil
Source: PLoS Negl Trop Dis. 2025 Feb 28;19(2):e0012880. doi: 10.1371/journal.pntd.0012880 (PMC11893133; doi:10.1371/journal.pntd.0012880)
Supplement: Attachment — Submitted filename: Validation_resubmission_Response to reviewers.docx [file pntd.0012880.s002.docx]

**Response to reviewer comments and questions**

**1. Reviewer #1:** The following comments need to be addressed by the authors. Page 6, please specify in more detail how the researchers make sure equal size of blood drops on each circle of the filter paper and what to do if the blood drop overlays to each other. If such DBS is meant to be used in limited resource area, shipping under room temperature should be tested.

**Authors’ response:** We used Whatman 903 filter paper cards for the DBS collection in our study. These cards feature five half-inch (12.7 mm) circles that each hold 75 to 80 μL of sample. Since the DBS collection was completed by trained nurses, they were experienced in preparing the participants for the collection, including allowing a full blood drop to form and fall naturally from the fingertip, rather than rushing the collection process by touching the finger to the card to transfer the blood prematurely, for example. This experience helped ensure sample quality control, including sufficient sample size and avoiding contamination or layering of blood spots.

We have added a few clarifications to the text on Page 6, including the maximum volume of the circles on the Whatman 903 cards, and mentioning that layering in blood drops was not accepted in our quality control process, since this inhibits estimation of sample volume. We would also like to note that since only three DBS punches of 6 mm were needed, as mentioned on Page 6, line 117, and consistent with other DBS protocols, this ensures consistency in the volume of eluted blood samples. The protocol was for a collection of three circles of approximately 12.7 mm to ensure that if there were a quality control issue with one circle, for example, it would still be possible to take three punches of 6 mm from the remaining two circles.

Regarding the comment on the transportation of samples under refrigeration vs. room temperature, we determined in our study setting, refrigerated transportation was feasible for both this validation study and the large cluster randomized controlled trial that was associated with it. A systematic review has found that for longer term storage, as would be required for the cluster randomized controlled trial, refrigeration should be used to avoid antibody degradation. Please see: Amini F, Auma E, Hsia Y, Bilton S, Hall T, et al. (2021) Reliability of dried blood spot (DBS) cards in antibody measurement: A systematic review. PLOS ONE 16(3): e0248218. <https://doi.org/10.1371/journal.pone.0248218>

By using refrigerated transportation and storage of samples, we also aimed to ensure a consistent study protocol for the cluster randomized controlled trial, in which blood samples were to be collected over a longer period due to the sample size of the study, thus resulting in variable “room temperature” conditions. Field and laboratory conditions for other studies would be expected to vary even more widely based on location, time of year, etc., so our use of refrigeration enables our results to be more easily compared with others. However, we have added a note in the discussion, on Page 17, that other validation studies may wish to vary this aspect of the protocol, depending on their needs and the accessibility of refrigeration.

**2. Reviewer #1:** In the discussion, the authors described the poor sensitivity of detecting IgG by indirect IgG ELISA. Although the overall agreement between serum and DBS is quite high, the poor sensitivity of IgG ELISA could potentially bias the results when treating the equivocals from serum or DBS as negative. It is recommended that the micro-neutralization assay should be used as the gold standard and determine how equivocals should be treated in order to obtain the true sensitivity and specificity.

**Authors’ response:** Thank you for this suggestion, we agree that PRNT or other neutralization assay approaches should ideally be used as the gold standard when possible, including in future validation studies such as ours. However, PRNT was considered prohibitively costly for our study, in terms of financial and other resources, since samples would have had to be transferred and tested in other laboratories. Therefore, our study protocol, developed based on the team’s expertise and the scientific literature, such as the reference given below, was to consider equivocal samples as negative to take a consistent and conservative approach, which would not bias DBS sensitivity. Given that there were only three equivocal results in this study, two of which were DBS samples from individuals whose matched serum samples were negative, and one of which was a serum sample from an individual whose matched DBS was positive, we feel that this approach was justified. However, in subsequent studies using ELISA assays for IgG detection (and in the absence of a gold standard, e.g. testing for SARS-CoV-2), we have sought to retest equivocal samples prior to considering them as negative, and consider this to be a preferable approach rather than immediate reclassification as negative.

Sample reference:

- Schnabel I, Schneitler S, Schüttoff T, Trawinski H, Lübbert C, Jassoy C. Diagnostic Specificity of Two Dengue Virus IgG ELISAs after Yellow Fever and Japanese Encephalitis Virus Vaccination. Trop Med Infect Dis. 2022;8(1):7. doi:10.3390/tropicalmed8010007

**3. Reviewer #1:** Page 7, the sentences to be more specific for the readers to understand. For example, Line 125, “undiluted DBS eluates were treated as a 1:25…..corresponding to approximately 10.5ul of serum that were eluted in 250ul of the eluent solution.” Should be …”serum that were diluted in 250ul of the diluent. Also, Line 129, the sentence “…1:2 and 1:4, to equate to 1:25… of the original blood sample” should be corrected as “of the serum” not blood. Only half of serum will be extracted from original blood sample. Also, Line 135, 100ul of each thawed serum diluted to 1:100 should be equivalent to 1:4 of DBS. The sentence should be corrected.

**Authors’ response:** Thank you very much for this close reading and these suggestions to improve the clarity and accuracy of the manuscript. We have made the changes accordingly (Pages 6-7). Please note that for the comment on the previous line 135, now lines 147-148, we have sought to clarify that serum diluted to 1:100 is indeed equivalent to 1:4 of DBS, but that the procedure being described here was applied to all four sample types (serum, DBS eluate non diluted, diluted 1:2 and diluted 1:4), and thus have retained the mention of each sample type in these corrected lines.

**4. Reviewer #2:** Objectives clearly stated.  Small but sufficient sample size, appropriate stats. Evidence of ethical approval presented.  Additional comments: Abstract: Check grammar.  E.g. ‘ELISA via’ ◊ ‘ELISA of’ and ‘DBS limited’ ◊ ‘DBS’ limited’.

**Authors’ response:** Thank you for these suggestions to improve the clarity of the abstract, we have made changes accordingly (Page 2).

**5. Reviewer #2:** Author summary: An informative summary and interpretation of results is provided.
Introduction: Suggest not to use the word ‘drastic’, perhaps ‘significant’ instead. Last sentence: should it read ‘optimal DBS elution protocol’ rather than ‘optimal DBS dilution’?

**Authors’ response:** Thank you for these suggestions. We have changed the wording in the second sentence of the introduction as suggested (Page 3), but have left the last sentence unchanged (Page 5, line 93) since our aim was to identify the optimal DBS dilution (among non-diluted, diluted 1:2, diluted 1:4) and this was the only aspect of the elution protocol which was varied.

**6. Reviewer #2:** Methods: First sentence check grammar. After collection both samples were transported in refrigerated containers and the stored at -20.  Why were the DBS samples refrigerated?  This is contrary to one of their stated advantages in the introduction.  Could this affect the external validity of your results?  I.e. if DBS are transported without refrigeration in field studies, would they perform as well?  ◊ You could address this in discussion section.

**Authors’ response:** Thank you, we have corrected the phrasing of the first sentence in the Methods section (Page 5). For your questions on sample transportation and storage under refrigeration, please see our response to comment #1 from reviewer #1, as this was addressed in the final paragraph of our response.

Additionally, we would not comment on the performance of DBS without refrigeration since we did not evaluate this in our study. However, we have addressed DBS transportation and storage in the discussion, mentioning that future validation studies could consider evaluating variable conditions depending on their context (Page 17).

**7. Reviewer #2:** Inspection of DBS quality.  How many were rejected?  Were any serums rejected because of evidence of haemolysis? This is important insight into field-suitability of both sample types.  Please see <https://can01.safelinks.protection.outlook.com/?url=https%3A%2F%2Fpubmed.ncbi.nlm.nih.gov%2F34850241%2F&data=05%7C02%7Claura.pierce%40umontreal.ca%7C3a4883a428e64ef398a808dcd0da2c67%7Cd27eefec2a474be7981e0f8977fa31d8%7C1%7C0%7C638614882673153204%7CUnknown%7CTWFpbGZsb3d8eyJWIjoiMC4wLjAwMDAiLCJQIjoiV2luMzIiLCJBTiI6Ik1haWwiLCJXVCI6Mn0%3D%7C0%7C%7C%7C&sdata=grwoJy2LsdKYTfinX5E%2BAtpjBDoIS2Pk8caBfWPrxSA%3D&reserved=0> where a significant number of blood spots were rejected.

**Authors’ response:** Fortunately, no DBS or serum samples had to be rejected from the study for quality control purposes, though they were all carefully assessed for quality concerns. We have added this detail in the results (Page 11, Line 213-214).

‘Clean and sterile’ ◊ ‘sterile’

**Authors’ response:** Thank you for pointing out this redundancy, we’ve made the change (Page 7).

**8. Reviewer #2:** ‘101 participants with 2 samples per person’ – does this mean the sample size requirement was actually 202?  Please clarify.  If it was 202 then sampling 101 individuals twice each is suboptimal, because each of the two datapoints cannot be considered independent from one another.  If each individual was tested twice, please clarify whether they were sampled twice (both methods), or whether just one sample were just double tested.  ◊ I now see from results that just 119 datapoints are analysed.  Please clarify ‘We aimed to recruit 112 potential 148 participants, assuming that 10% of participants would not be eligible’ – they would not be recruited if they were not eligible.. Suggest using ‘screen’ instead of ‘recruit’ in this sentence.

**Authors’ response:** Thank you for these suggestions, we have rephrased the sample size requirements according to your suggestions to ensure clarity (Page 8).

**9. Reviewer #2:** Why was it decided that equivocal results would be treated as negative?  Was this decided a-priori or after reviewing results?  How would results be affected if the opposite decision was taken?

**Authors’ response:** Thank you for these questions, and please see our response to comment #2 above. In response to the additional concerns raised here, we have clarified in the manuscript that our decision to consider equivocal results as negative was made a-priori (Page 8). This was based on existing scientific literature as well as a desire to take a conservative approach and avoid introducing bias to sensitivity measurements.

As shown in Table 2, and discussed on Page 14, there were just three equivocal samples in our study, which were among the four discordant results between the 119 matched serum and DBS samples, if we consider initial results with all three possible classifications of positive, negative, and equivocal. Two DBS samples were found to be equivocal, and thus treated as negative, and the corresponding serum samples were negative. One DBS sample was positive, and the corresponding serum sample was found to be equivocal, and thus treated as negative. Due to the rarity of either DBS or serum samples being equivocal, using an alternate decision (i.e. treating equivocal samples as positive) would have had little effect on the results, in which 115 of 119 matched samples (97 %) showed unequivocally concordant results.

**10. Reviewer #1:** Page 4, Line 65, the sentence of “Seroprevalence studies commonly use DENV IgG antibodies to identify previous dengue infection…..” should be rephrased since a four-fold increase in IgG antibody titers from the sequentially collected serum samples can also be regarded as acute infection.

**Authors’ response:** Thank you for this comment, we have adjusted the phrase accordingly (Page 4).

**11. Reviewer #1:** Page 5, line 91, the sentence of “The study design was cross-sectional study was conducted…” should be corrected in grammar.

**Authors’ response:** Thank you, as mentioned in response to comment #6 above, we have corrected the phrasing of the first sentence in the Methods section (Page 5).

**12. Reviewer #1:** Table 3, p-value is not 0. Please follow the scientific usage of decimals or just specify <0.05 or <0.01 or <0.001

**Authors’ response:** Thank you for pointing this out, it was indeed a rounding error that we have corrected (Page 12).

**13. Reviewer #1:** Page 13, line 210-212, it is better to illustrate what the acceptable range is. Based on the discussion, Line 229, if the acceptable intra or inter assay COV were acceptable under the range of 10%, then it should be stated in Line 212, the COV are all lower than 10%, instead of saying 15% as it is within 5% in fact in Table 4. Also, Table 4, the sample size of each tercile should be illustrated.

**Authors’ response:** Thank you for this suggestion, we have clarified that 10% and 15% are the acceptable limits to provide more context to readers for the interpretation of the results. We have also clarified the sample sizes in Table 4, including the overall sample size (n=30) and the sample size for each tercile (n=10) (Page 13).

**14. Reviewer #2:** Analysis presented matches plan and results are generally well presented.  Additional comments: Mean age was 22.3 years.  Can you give any further details of the participants, for example their country of birth and/or whether they grew up in a dengue-endemic country (e.g. Brazil). Since they are not young children (which most serological surveillance studied focus on), it is important to try to understand the likelihood of multiple historic dengue infections (where IgG titres will be high) vs. only one (where IgG titres will be low), among the positives.  Either way, this potential limitation should be picked up in the discussion: Are the findings applicable to younger populations where titres may be low? ◊ I now see that this is well addressed in discussion section already.

**Authors’ response:** Thank you for revising your comment after reviewing the discussion. We would like to offer the additional clarification that the questionnaire did not assess country of birth and/or long-term residence in a dengue-endemic region. However, participants were asked about their history of dengue episodes, and we discussed the potential impact of this upon the results (for example, Page 14-15 and lines 274-285).

**15. Reviewer #2:** Please provide a more detailed explanation of figure 2.  What are the horizontal dotted lines? Do you need to comment on the distribution of data points also – lower IVs are generally below, and higher IVs are generally above.  Does this mean that DBS measurement is bias in one direction at lower concentrations, but the other direction at higher?  Please explain in more detail.

**Authors’ response:** Thank you for this comment, we have clarified in the figure title that the horizontal dotted lines represent the 95% CIs (please see Page 13, line 244, note that this is now Figure 4). For the second part of your comment, we also request that you review our discussion of this plot (Page 14 line 267-271), where we’ve mentioned that at index values lower than about 1.5, we found that DBS samples tended to have a higher signal than the matched serum sample, but at index values above 1.5, mean differences were more equally distributed.

**16. Reviewer #2:** Inter- and intra- assay correlation coefficients are presented. Please define these terms in more detail.  Also, I am not totally clear which data these are based on.  For example, do these calculations include the primary data, where all 119 were tested, or just the re-test data, where 30 positives were retested?

**Authors’ response:** Thank you for pointing this out. We have provided additional information on how the inter- and intra-assay correlation coefficients were calculated and what these represent (Page 9, lines 195-204). We have also clarified there and on Page 13 that the sample for these calculations were based on the 30 retested positives (10 from each of the 3 terciles).

**17. Reviewer #2:** Conclusions are well supported by the data.  Limitations acknowledged.  Useful study in this field. Fair interpretation of results.  Some of my comments above may be better addressed in the discussion section. In limitations, you could mention that only the dilution step in your elution protocol was varied.  Is it possible that varying other steps could have yielded better results?  In particular, I wonder if shaking at 300rpm overnight could have disrupted proteins and affected IgG binding.

**Authors’ response:** Thank you for this suggestion, and we have added a comment in the discussion section (Page 17) to acknowledge that we varied only the dilution step and other validation studies may wish to examine other questions. Regarding the choice to shake at 300 rpm overnight, this approach was favored to ensure adequate elution, i.e. extraction of the dried blood from the blotter. Based on the experience of the laboratory team, there was no reason to expect that this slow overnight agitation would limit or affect the binding of IgG antibodies, and there are many studies in the literature that have taken similar protocols. For examples, please see:

- Ross RS. et al. Detection of infections with hepatitis B virus, hepatitis C virus, and human immunodeficiency virus by analyses of dried blood spots—performance characteristics of the ARCHITECT system and two commercial assays for nucleic acid amplification. Virol J. 10(1), https://doi.org/10.1186/1743-422X-10-72 (2013).
- Louie KS. et al. Evaluation of Dried Blood Spots and Oral Fluids as Alternatives to Serum for Human Papillomavirus Antibody Surveillance. mSphere. 3(3), <https://doi.org/10.1128/mSphere.00043-18> (2018).
- Holguín A. et al. Dried blood as an alternative to plasma or serum for Trypanosoma cruzi IgG detection in screening programs. Clin Vaccine Immunol. 20(8), 1197–1202, https://doi.org/10.1128/CVI.00221-13 (2013).
- Kumar A, Mhatre S, Dikshit R. Utility of dried blood spots in detecting helicobacter pylori infection. Indian J Med Microbiol. https://doi.org/10.4103/ijmm.IJMM_19_441 (2019).

**18. Reviewer #1:** In the discussion, the authors described the poor sensitivity of detecting IgG by indirect IgG ELISA. Although the overall agreement between serum and DBS is quite high, the poor sensitivity of IgG ELISA could potentially bias the results when treating the equivocals from serum or DBS as negative. It is recommended that the micro-neutralization assay should be used as the gold standard and determine how equivocals should be treated in order to obtain the true sensitivity and specificity.

**Authors’ response:** Thank you, we agree that micro-neutralization or plaque reduction neutralization assays should be used in future validation studies as the gold standard when feasible, and we have added a comment to more explicitly address this in our discussion (Page 16, lines 316-318). We also responded to a similar question from reviewer 2 above (please see comment #2).

**19. Reviewer #2:** Thank you for inviting me to review this manuscript.  Overall this is a well conducted and well presented study.  The authors should be commended for doing this pragmatic research which will help others working in this field.

**Authors’ response:** Thank you to both reviewers for your comments, which helped us to improve the clarity of the manuscript.
